# Functional combinatorial precision medicine for predicting and optimizing soft tissue sarcoma treatments

**DOI:** 10.1038/s41698-025-00851-7

**Published:** 2025-03-22

**Authors:** Sharon Pei Yi Chan, Masturah Bte Mohd Abdul Rashid, Jhin Jieh Lim, Janice Jia Ni Goh, Wai Yee Wong, Lissa Hooi, Nur Nadiah Ismail, Baiwen Luo, Benjamin Jieming Chen, Nur Fazlin Bte Mohamed Noor, Brandon Xuan Ming Phua, Andre Villanueva, Xin Xiu Sam, Chin-Ann Johnny Ong, Claramae Shulyn Chia, Suraya Zainul Abidin, Ming-Hui Yong, Krishan Kumar, London Lucien Ooi, Timothy Kwang Yong Tay, Xing Yi Woo, Tan Boon Toh, Valerie Shiwen Yang, Edward Kai-Hua Chow

**Affiliations:** 1 Cancer Science Institute of Singapore, National University of Singapore, 14 Medical Drive, #12-01 Centre for Translational Medicine, Singapore, 117599 Republic of Singapore; 2 KYAN Technologies, 1 Research Link, #05-45, Singapore, 117604 Republic of Singapore; 3 Bioinformatics Institute, Agency for Science, Technology and Research (A*STAR), 30 Biopolis Street, #07-01 Matrix, Singapore, 138671 Republic of Singapore; 4 NUS Centre for Cancer Research (N2CR), Yong Loo Lin School of Medicine, National University of Singapore, 14 Medical Drive, #12-01 Centre for Translational Medicine, Singapore, 117599 Republic of Singapore; 5 The Institute for Digital Medicine (WisDM), Yong Loo Lin School of Medicine, National University of Singapore, 28 Medical Drive, #05-COR, Singapore, 117456 Republic of Singapore; 6 The N1 Institute for Health, National University of Singapore, 28 Medical Drive, Singapore, 117456 Republic of Singapore; 7 Translational Precision Oncology Laboratory, Institute of Molecular and Cell Biology (IMCB), Agency for Science, Technology and Research (A*STAR), 61 Biopolis Drive, Proteos, Singapore, 138673 Republic of Singapore; 8 Division of Medical Oncology, National Cancer Centre Singapore, 30 Hospital Boulevard, Singapore, 168583 Republic of Singapore; 9 Department of Anatomical Pathology, Singapore General Hospital, College Road, Level 7 Academia, Singapore, 169856 Republic of Singapore; 10 Laboratory of Applied Human Genetics, Division of Medical Sciences, National Cancer Centre Singapore, 30 Hospital Boulevard, Singapore, 168583 Republic of Singapore; 11 Department of Sarcoma, Peritoneal and Rare Tumours (SPRinT), Division of Surgery and Surgical Oncology, National Cancer Centre Singapore, 30 Hospital Boulevard, Singapore, 168583 Republic of Singapore; 12 Oncology Academic Clinical Program, Duke-NUS Medical School, 8 College Road, Singapore, 169857 Republic of Singapore; 13 SingHealth Duke-NUS Surgery Academic Clinical Program, Duke-NUS Medical School, 8 College Road, Singapore, 169857 Republic of Singapore; 14 Department of Orthopaedic Surgery, Singapore General Hospital, 10 Hospital Boulevard, Tower Level 4 SingHealth Tower, Singapore, 168582 Republic of Singapore; 15 Department of Neurology, National Neuroscience Institute (Singapore General Hospital Campus), Outram Rd, Singapore, 169608 Republic of Singapore; 16 Department of Neurosurgery, National Neuroscience Institute (Singapore General Hospital Campus), Outram Rd, Singapore, 169608 Republic of Singapore; 17 Hepato-pancreato-biliary and Transplant Surgery, Singapore General Hospital, Outram Rd, Singapore, 169608 Republic of Singapore; 18 Department of Pharmacology, Yong Loo Lin School of Medicine, National University of Singapore, 16 Medical Drive, Singapore, 117600 Republic of Singapore; 19 Department of Biomedical Engineering, College of Design and Engineering, National University of Singapore, 4 Engineering Drive 3, #04-08, Singapore, 117583 Republic of Singapore

**Keywords:** Sarcoma, Sarcoma, Phenotypic screening

## Abstract

Soft tissue sarcomas (STS) are rare, heterogeneous tumors with poor survival outcomes, primarily due to reliance on cytotoxic chemotherapy and lack of targeted therapies. Given the uniquely individualized nature of STS, we hypothesized that the ex vivo drug sensitivity platform, quadratic phenotypic optimization platform (QPOP), can predict treatment response and enhance combination therapy design for STS. Using QPOP, we screened 45 primary STS patient samples, and showed improved or concordant patient outcomes that are attributable to QPOP predictions. From a panel of approved and investigational agents, QPOP identified AZD5153 (BET inhibitor) and pazopanib (multi-kinase blocker) as the most effective combination with superior efficacy compared to standard regimens. Validation in a panel of established patient lines and in vivo models supported its synergistic interaction, accompanied by repressed oncogenic MYC and related pathways. These findings provide preliminary clinical evidence for QPOP to predict STS treatment outcomes and guide the development of novel therapeutic strategies for STS patients.

## Introduction

Soft tissue sarcomas (STS) are rare mesenchymal neoplasms with diverse clinical and molecular features. Representing less than 1% of all cancers, STS reflects the highest incidence among rare malignancies and disproportionately accounts for 20% of pediatric and young adult tumors^1–3^. Treating STS presents several challenges due to their rarity, diverse nature and contrasting responses to conventional therapies^4^. Comparative genetic analysis has highlighted varying frequencies of chromosomal translocations, oncogenic mutations and gene amplifications across all STS histologies^5,6^. This genomic and biologic complexity contributes to divergent histomorphologies and tumor-specific treatment responses. Approximately half of newly diagnosed STS patients eventually progress to advanced stages and 40% eventually succumb to the disease^7^. There is therefore an urgent need to improve treatment outcomes across the different histologic and molecular subtypes in STS.

Current treatments for STS often involve a multimodal approach, including surgery, radiation, and chemotherapy. Even with adequate locoregional control, nearly 40% of patients develop metastatic disease^8^. Cytotoxic chemotherapy is the cornerstone in the advanced setting, with single agent anthracycline (e.g., doxorubicin) as the preferred first-line standard of care for most subtypes of metastatic or inoperable STS. Despite decades of extensive clinical trials, anthracyclines, either alone or in combination with ifosfamide, remain the most effective and widely used systemic treatment for STS^9–12^. Yet, its use is associated with a dismal overall response rate of less than 20%, and poor median overall survival of approximately a year across all histologies^13^. Cumulative dose-dependent cardiotoxicity further limits its use^14^. Beyond the first-line setting, gemcitabine-based combinations, pazopanib, eribulin and trabectedin are approved treatment options in refractory/relapsed STS, but toxicity is high with dismal response rates of 4–10% and a median progression-free survival of 4 months^15–18^. Despite decades of research, patients with STS continue to face dismal survival rates, as effective therapies and salvage options remain limited^19^. Furthermore, because of the rarity and diverse biology of STS subtypes, there are no large-scale prospective clinical trials or real world evidence datasets across STS to guide treatment. A personalized, multidisciplinary treatment strategy may offer improved outcomes for patients with soft tissue sarcoma. To this end, precision medicine strategies to predict patient response or resistance to treatments, and identifying novel therapeutics effective for patients who do not respond to existing options are indispensable to making significant advances.

Functional precision oncology (FPO) has promising potential to expand treatment options when standard or salvage options are exhausted^20–24^. These platforms are especially valuable for rare cancers such as STS constrained by the scarcity of clinical trial data, yet unsuited for genomic-guided precision approaches given the lack of actionable driver mutations. Moreover, these strategies often involve comprehensive drug assessment from a repertoire of FDA-approved and investigational agents on patient-derived avatars, with FPO recommendations delivered to clinicians within clinically actionable timeframes. We have previously demonstrated in a 71-patient relapsed/refractory non-Hodgkin’s lymphoma clinical study that a functional precision medicine platform can effectively guide treatment, with several exceptional responders^25^. This ex vivo drug sensitivity platform, quadratic phenotypic optimization platform (QPOP), has also been successfully applied preclinically^26–29^ and clinically^30^ to identify effective combinations against a range of drug resistant and relapsed/refractory cancers. By leveraging on small experimental datasets, QPOP analyzes a predesigned array of 155 test combinations performed on a primary patient sample to rank and compare all possible therapeutic combinations from a 12-drug set. Given the highly heterogenous nature of STS and lack of actionable mutations and common molecular targeted therapies in most STS, we hypothesize that application of QPOP in primary STS patient samples can improve identification of patient and subtype-specific drug combinations. In this study, we applied an ex vivo QPOP protocol to evaluate the drug response profile across 45 primary STS patient samples using a panel of FDA-approved and investigational agents. We then performed concordance analysis to assess the discriminative ability of QPOP in predicting treatment outcomes, and showcased its feasibility and actionability in either QPOP-guided or retrospective clinical studies. We also explored QPOP’s ranking function for combination discovery and reported the in vitro and in vivo therapeutic potential of a novel epigenetic-based pairing that may outperform current standard of care. Taken together, by utilizing a patient-based drug sensitivity analytic approach, we demonstrated the clinical feasibility of QPOP to identify patient-specific therapeutic sensitivities and elucidated the cellular mechanisms underlying a novel, top-ranked targeted drug combination that provides scientific rationale for further clinical development.

## Results

### QPOP results are concordant with clinical outcomes

**Fig. 1:**
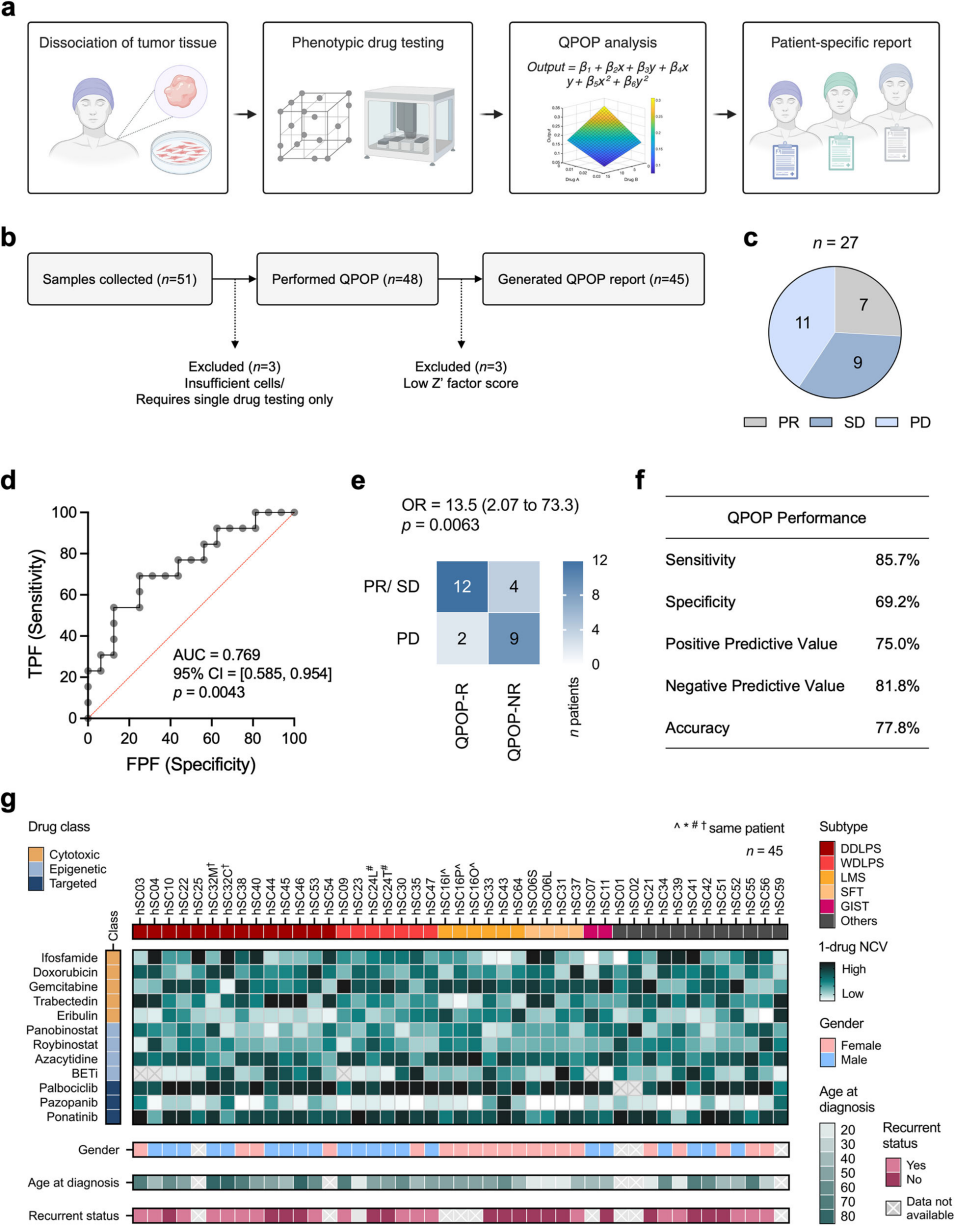
Summary of QPOP study and its clinical application in soft tissue sarcomas. **a** Schematic overview of QPOP workflow, from sample collection and drug panel selection to generation of patient-specific QPOP report. **b** CONSORT diagram of the number of patient samples collected and the resulting 45 QPOP drug sensitivity data generated. **c** Pie chart depicting proportion of treatment responses achieved from a total of 14 evaluable patients. PR partial response, SD stable disease, PD progressive disease. **d** Receiver operating characteristic (ROC) curve illustrating QPOP’s discriminative ability. *P*-value was determined using Wilcoxon-Mann-Whitney test. **e** Contingency table of response cohorts based on defined QPOP outcomes. QPOP-R QPOP-defined responder, QPOP-NR QPOP-defined non-responder. *P*-value was determined by Fisher’s exact test. **f** Table showing QPOP’s performance in clinical settings based on the defined outcomes. **g** Summary of QPOP one-drug rankings in individual samples clustered according to STS subtype and corresponding clinical characteristics (gender, age at first diagnosis, and recurrent status). A smaller rank value represents greater cell killing.

To determine the clinical accuracy of QPOP’s drug sensitivity predictions, we performed concordance analysis by comparing QPOP-defined outcomes with clinical outcomes from either QPOP-guided therapies or prior lines of treatment. Briefly, fresh tumor biopsies or resections were sampled from consented patients and QPOP ex vivo drug treatment was performed. QPOP results were analyzed and consolidated as a personalized report for each patient. This process is graphically summarized in Fig. 1a. During this study period, we collected a total of 51 samples. Three out of 51 (5.88%) cases were excluded due to insufficient sample material, while another three cases were excluded due to not fulfilling quality control requirements based on Z’ scores. Overall, QPOP reports were successfully generated for 45 of 51 (88.2%) primary patient samples (Fig. 1b). Among 27 distinct treatment outcomes from 14 evaluable patients, 7 (25.9%) were classified as partial response (PR), 9 (33.3%) as stable disease (SD) and the remaining 11 (40.7%) as progressive disease (PD) using RECIST 1.1 criteria^31^ (Fig. 1c). Mean normalized cell viability (NCV) refers to the average NCV of all possible permutations of a single combination or therapy. To stratify treatment outcomes as QPOP-defined responders or non-responders, the cut-off for NCV was defined at 0.8305 based on the receiver operating characteristic (ROC) curve (Fig. 1d). The ROC curve had an AUC value of 0.769 with a 95% confidence interval from 0.585 to 0.954. With a *P*-value of < 0.05, the AUC_ROC_ was significant, suggesting a fair and meaningful test based on the defined cut-off and the platform’s ability to discriminate between positive and negative cases accurately. Using this cut-off, we compared 14 responder (QPOP-R) cases with 13 non-responder (QPOP-NR) cases and noted a significantly higher frequency for PR/SD status for treatment cases in which QPOP defined to be responders as compared to non-responders (odds ratio (OR) of PR/ SD for the QPOP responder group was 13.5; confidence interval (CI) 2.07–73.3; *p* = 0.0063; Fig. 1e). Clinical concordance analysis showed QPOP had a total predictive value (TPV) of 77.8% (Fig. 1f). High concordance between observed and predicted outcomes shows the potential for QPOP to inform treatment response in STS.

To compare the differential drug sensitivities of individual patient samples, mean NCV for each drug was plotted in a heatmap (Fig. 1g). A lower NCV indicates greater cell killing. The 12-drug panel comprising STS standard of care, FDA approved drugs and promising investigational drugs and their respective dosages were kept constant across all samples. This allowed evaluation of relative sensitivities against overall cohort with culminative QPOP data. BETi collectively refers to the compounds OTX015 or AZD5153, unless otherwise specified. Due to the reported dose limiting toxicities of OTX015^32^, OTX015 was subsequently replaced by AZD5153, a bivalent BETi, in our panel to ensure clinical relevance^33^. Generally, most samples displayed sensitivity towards second-line agent pazopanib and mixed response towards first-line anthracycline doxorubicin and alkylating agent ifosfamide. Within the limitation of a small test group, we did not observe any subtype-dependent patterns of sensitivity.

### QPOP can effectively guide treatment in patients with limited salvage options

**Fig. 2:**
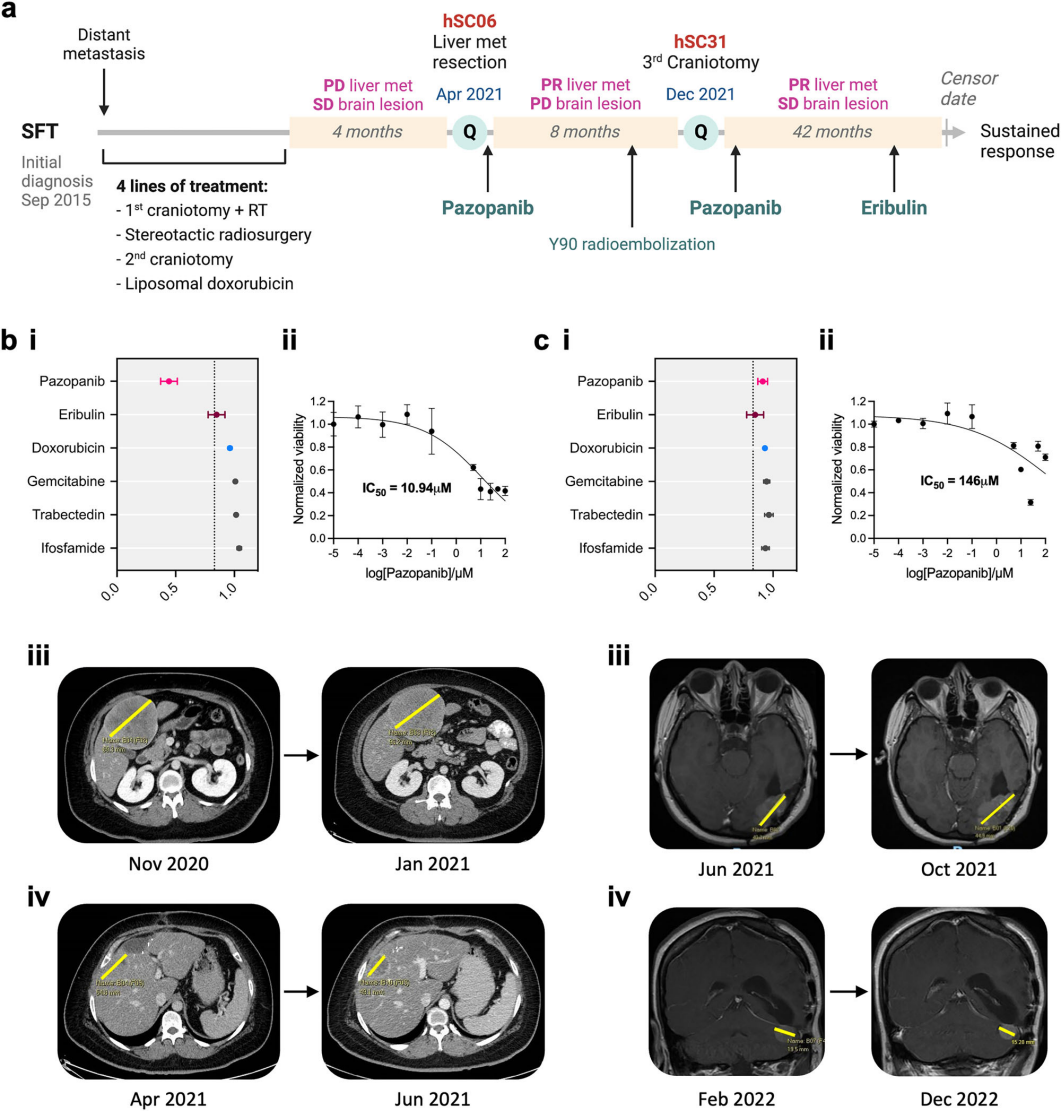
QPOP to guide treatment response in an SFT patient. **a** Treatment timeline for the patient. The green circle denotes the time point at which QPOP analysis was performed. **b** (i) Forest plot depicting QPOP-derived normalized cell viability (NCV) scores (mean ± SD) of top-ranked single agents in comparison to other standard therapies. (ii) Single-drug dose-response curve of pazopanib in hSC06. (iii) Computed tomography (CT) imaging of the dominant liver metastasis progressing on liposomal doxorubicin and (iv) before and after pazopanib treatment. **c** (i) Forest plot depicting QPOP-derived normalized cell viability (NCV) scores (mean ± SD) of top-ranked single agents in comparison to other standard therapies. (ii) Single-drug dose-response curve of pazopanib in hSC31. (ii) Magnetic resonance imaging (MRI) of the locally recurrent primary brain lesion progressing on pazopanib and (iv) before and after treatment with eribulin.

QPOP-guided therapies were used as off-label treatments in a subset of patients who exhausted standard of care options. We highlight here an exceptional case in the treatment of an aggressive solitary fibrous tumor (SFT) which has failed multiple lines of treatment. A young female in her 20 s initially with brain-limited cerebroventricular SFT, developed high volume distant metastasis to bone and liver (Fig. 2a). The patient was treated with standard of care liposomal doxorubicin, through which her disease progressed, with increase in size of her dominant liver metastatic lesion. She subsequently underwent a palliative resection of this lesion. A portion of the resected tumor, referred to as hSC06, was dissociated in our laboratory and used for ex vivo drug testing. QPOP suggested a lack of sensitivity towards doxorubicin (Fig. 2bi), recapitulating disease progression through doxorubicin in the liver lesion (increase in size from 8.9 to 9.6 cm; Fig. 2biii). QPOP also identified single agent pazopanib to be most effective in hSC06, outperforming other standard regimens in mean NCV, and corroborated by dose response assay (Fig. 2bi-ii). Following her recovery from surgery, she was commenced on palliative pazopanib as second line systemic treatment. Repeat imaging two months later showed response in her liver lesions (decrease in size from 5.5 to 2.8 cm; Fig. 2biv), but continued progression in her brain lesions, with the largest lesion measuring 4.5 cm in size. The patient underwent a craniotomy in December 2021 and a portion of the resected tumor was used for ex vivo drug testing with same drug panel. QPOP identified increased resistance towards pazopanib, corroborated by more than 10-fold increase in IC_50_, and ranked eribulin to be most effective in hSC31 (Fig. 2ci-ii). This recapitulated what was seen clinicoradiologically: continued response in the liver lesions but progression in the recurrent brain lesion with pazopanib treatment (increase in size from 4.0 to 4.5 cm; Fig. 2ciii). Given the shift towards eribulin sensitivity in hSC31, the patient was therefore given eribulin in May 2022 to treat her brain disease, which may also control her disease systemically. Intriguingly, this successfully arrested disease progression in the brain (decrease in size from 2.0 cm to 1.5 cm; Fig. 2civ), while maintaining stable disease extracranially (further reduction to 2.8 cm for liver lesion; Supplementary Fig. 1). The patient remains well and fully functional at more than 2 years since commencement of eribulin, and has far outlived her prognosis of one year. This results provide important proof-of-concept that QPOP can not only accurately predict drug response, but also efficiently identify non-conventional, clinically beneficial therapies in ultra-rare cancers with limited therapeutic options.

**Fig. 3:**
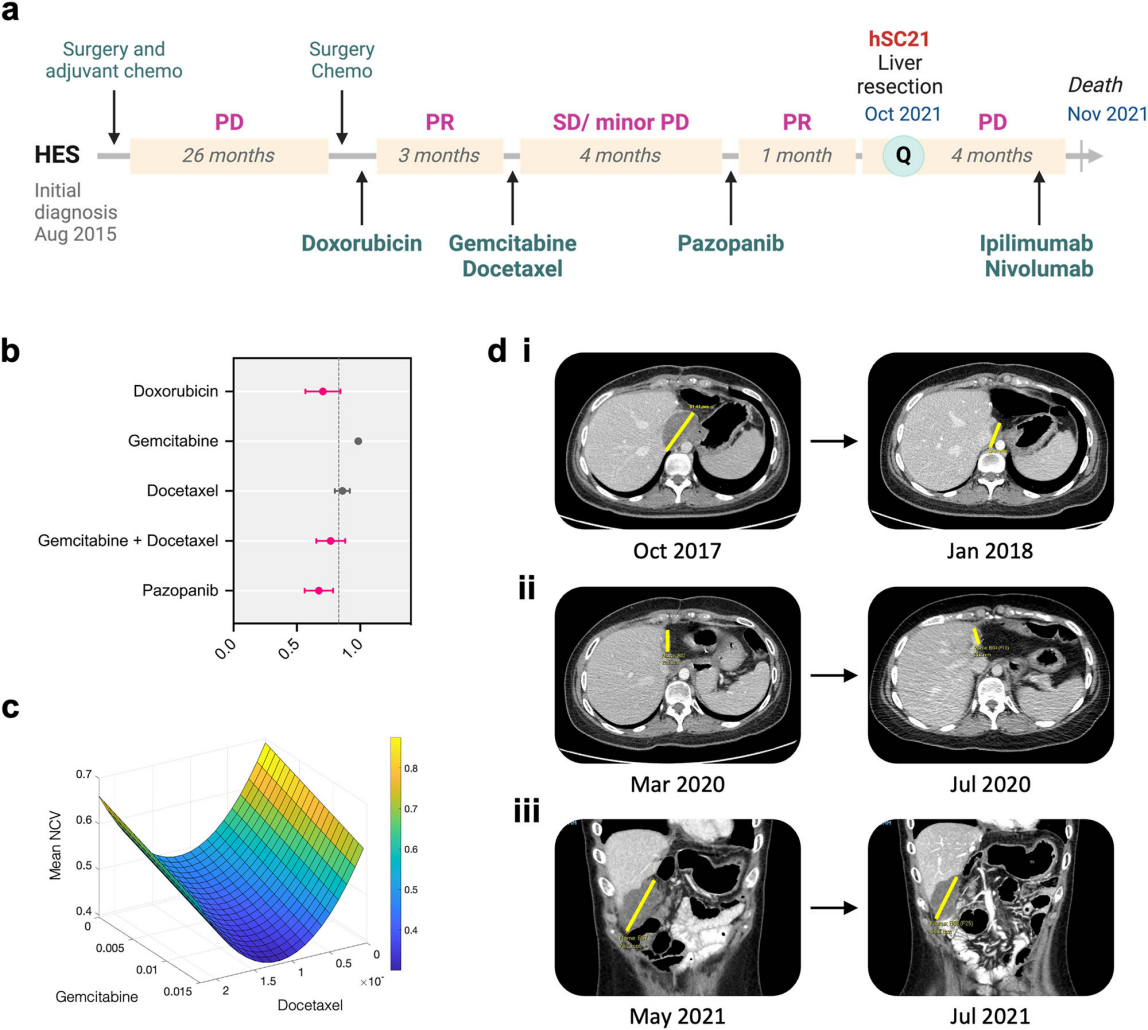
Retrospective case analysis correlating treatment response with QPOP-defined outcomes in a HES patient. **a** Treatment timeline for the patient. The green circle denotes the time point at which QPOP analysis was performed. **b** Forest plot depicting QPOP-derived normalized cell viability (NCV) scores (mean ± SD) of single and combination agents in prior lines of treatment. **c** Parabolic response surface map of drug interaction between gemcitabine and docetaxel. **d** Computed tomography (CT) imaging of the liver lesion before and after treatment with (i) doxorubicin, (ii) gemcitabine and docetaxel, and (iii) pazopanib treatment.

In another example of concordance between QPOP-predicted treatment sensitivity and positive clinical outcome, a young female in her 20 s with hepatic embryonal sarcoma (HES) was referred for QPOP ex vivo drug testing when her disease relapsed following initial response towards multiple lines of treatment with standard single and combination regimens (Fig. 3a). QPOP identified sensitivity toward single-agents doxorubicin and pazopanib, as well as the combination of gemcitabine and docetaxel (Fig. 3b). Sensitivity towards gemcitabine and docetaxel was further supported by the parabolic response surface map (RSM) depicting increasing drug interaction with increasing dose of either agent at specific dose range (Fig. 3c). These results recapitulated the observed clinical response of the patient: partial response to doxorubicin (6.1 to 3.3 cm; Fig. 3di), stable disease or modest response with gemcitabine and docetaxel (2.6 to 2.0 cm; Fig. 3dii) and partial response toward pazopanib (7.6 to 6.5 cm; Fig. 3diii). Following 4 months of response to pazopanib, the patient eventually progressed. The patient declined further ex vivo drug testing, was commenced on ipilimumab and nivolumab and subsequently relapsed.

### QPOP identified BETi/pazopanib as most frequently top-ranked across all primary STS patient samples

**Fig. 4:**
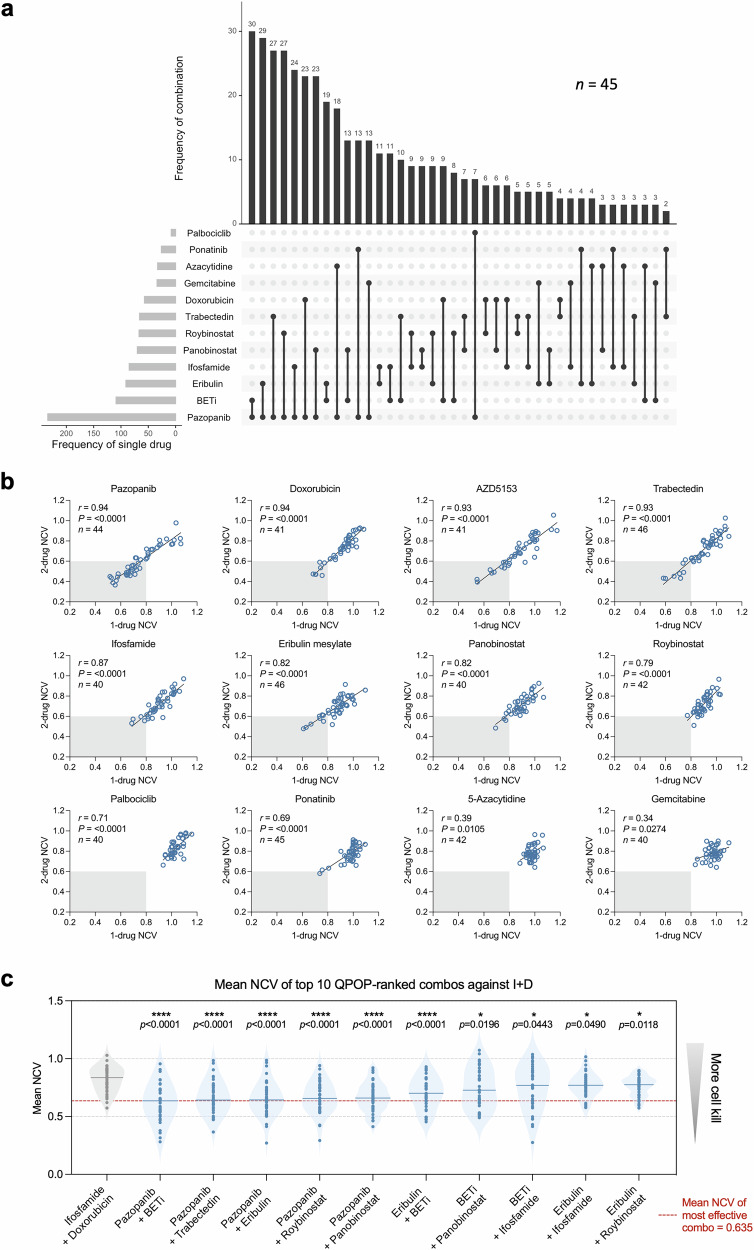
Pazopanib + BETi is the most frequently occurring drug pair demonstrating the strongest combinatorial efficacy across all 45 primary STS patient samples. **a** Frequency at which individual drugs and combinations appear within the top-10 unique two-drug combinations were plotted in an upset plot across all PDCs (*n* = 45). “Frequency of combination” refers to the number of times a drug pairing appeared within the top ten two-drug combinations across all PDCs. “Frequency of single drug” refers to the total number of times each drug has appeared within any of the top ten ranked combination. **b** Scatter plots showing correlation between one-drug and two-drug NCVs. Pearson correlation coefficient (*r*), *p*-, and *n*-values shown represent correlation analysis for the overall cohort. **c** Summary of mean NCV for top-ranked drug combinations against standard of care ifosfamide and doxorubicin. All statistical analyses were performed using two-tailed Student’s *t* test; *, *P* < 0.05; ****, *P* < 0.0001 compared to ifosfamide + doxorubicin.

While QPOP is primarily applied as a functional precision medicine platform to obtain patient-specific information, we also explored QPOP’s ranking function to detect recurrent sensitivity patterns in our STS cohort that may indicate previously unexplored but effective combinations. Analysis of top 10 unique two-drug combos across all 45 samples revealed high frequencies of pazopanib-based combinations, commonly paired with BETi, eribulin, trabectedin or roybinostat (Class II selective HDACi). Pazopanib and BETi combination occurred as top-ranked in 30 out of 45 (66.7%) samples, whereas standard of care ifosfamide and doxorubicin occurred in only 6 out of 45 (13.3%) samples (Fig. 4a). As single-drug sensitivity testing is traditionally used as a predictive measure of therapeutic response, we additionally performed ex vivo single-drug dose response assay for each of the 12 drugs in parallel to QPOP drug treatment across all samples (Supplementary Fig. 2). We observed nominally positive correlation between single-drug sensitivity and QPOP median rank in ifosfamide and doxorubicin, but not in targeted agents (Supplementary Fig. 3). This result suggests that single-drug sensitivity alone is not a reliable screen to inform combination efficacy and therefore QPOP represents an unbiased platform for the systematic identification of optimal drug combinations.

To assess if individual drug action is enhanced in combination, we analyzed the relationship between QPOP-derived one-drug mean NCV values and two-drug NCV values from top 20 combinations of each sample. Interestingly, strong positive correlation was observed for clinically approved first- and second-line therapies like pazopanib, doxorubicin, trabectedin and ifosfamide, as well as for promising investigational agent AZD5153. These drugs are also frequently top-ranked individually and/or in combination. Other drugs such as 5-azacytidine and gemcitabine, did not, however, exhibit a correlation between one-drug and two-drug viability outputs (Fig. 4b). This suggests that some drugs, when paired with the right combinatorial partner, exhibits stronger gain in efficacy over single agents, whereas others drugs are limited to single-agent efficacy. Overall, this analysis highlights the potential of certain drugs to be more effective as dual therapy and provide insights into critical drug targets in the context of STS.

To further explore the degree of combinatorial efficacy in top-ranked drug pairings, we plotted mean NCV of each drug pair and compared against that of standard of care, ifosfamide and doxorubicin (Fig. 4b). Across the top-10 pairings, pazopanib and BETi demonstrated the lowest mean NCV score, indicative of highest cell kill. As epigenetic dysregulation is increasingly recognized as an important contributor in the pathogenesis of STS^34,35^, there has been growing interest in emerging pharmacologic strategies to study and therapeutically target epigenetic regulatory proteins^36^. Therefore, we decided to further investigate the efficacy of pazopanib and BETi in STS, over chemotherapy-based combinations where toxicity is a major limitation^37^. Mean NCV for pazopanib-BETi pairing across all samples was 0.635, demonstrating a 18.4% increase in killing compared to ifosfamide and doxorubicin at 0.819. Strong combinatorial efficacy demonstrated by pazopanib-BETi therefore suggests common targetable vulnerabilities across STS subtypes.

### Pazopanib and BETi demonstrate synergistic activity superior to standard of care ifosfamide and doxorubicin

**Fig. 5:**
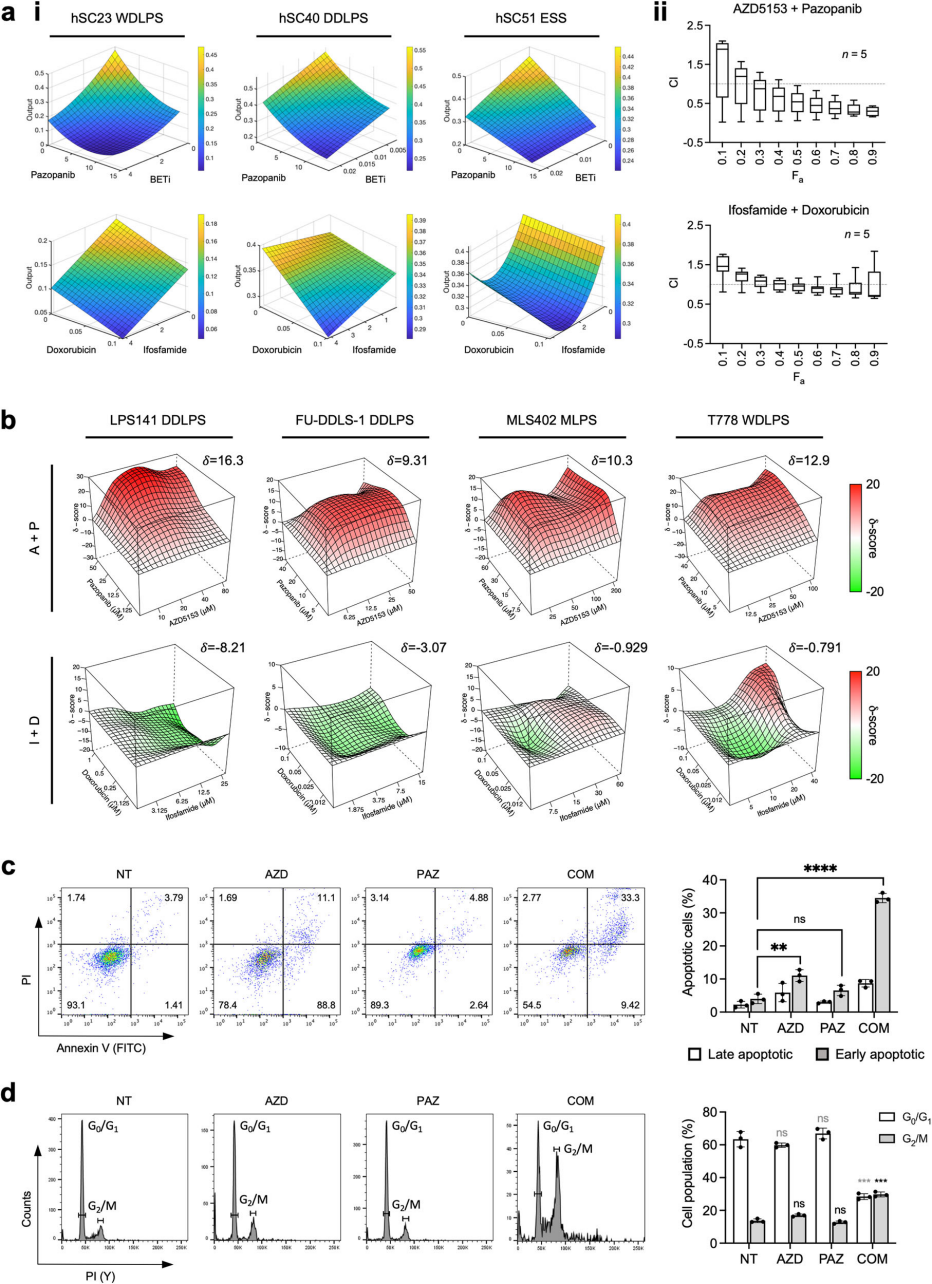
Pazopanib and BETi interact synergistically and demonstrate anti-proliferative and pro-apoptotic activity. **a** (i) QPOP-derived parabolic response surface maps (RSMs) illustrating drug interactions of top-ranked pazopanib + BETi (top panel) compared to standard of care ifosfamide and doxorubicin (bottom panel) in three representative samples. WDLPS, well-differentiated liposarcoma; DDLPS de-differentiated liposarcoma, ESS endometrial stromal sarcoma. (ii) Range of Pazopanib + BETi and ifosfamide + doxorubicin combination index (CI) values for five PDSCs plotted against fraction of cells killed, *F*_a_. **b** 3D synergy distribution derived from Bliss independence model charts synergistic (red) or antagonistic (green) dose regions for pazopanib + AZD5153 or ifosfamide + doxorubicin in cell lines of four subtypes using inhibition (%) of cell viability as phenotypic readout. A + P, AZD5153 + Pazopanib; I + D, Ifosfamide + Doxorubicin. MLPS myxoid liposarcoma. **c** Annexin V/PI staining and quantification following 48 h drug treatment in LPS141. Data presented as mean ± SD, *n* = 3. **d** Cell cycle distribution analysis showing percentage of cells in G0/G1 and G2/M phases following 24 h drug treatment in LPS141. Data presented as mean ± SD, *n* = 3 biological replicates. Asterisks in black and gray represent statistical comparisons to NT (untreated) within G0/G1 and G2/M phases, respectively. **, *P* < 0.01; ***, *P* < 0.001; ****, *P* < 0.0001 compared to NT controls.

We further evaluated the drug-drug interaction between top-ranked pazopanib and BETi, and compared it against standard of care ifosfamide and doxorubicin in three representative patient samples (Fig. 5a). Parabolic RSM generated via the QPOP algorithm demonstrated the synergistic interaction between pazopanib and BETi, as depicted by the decreasing cell viability output with increasing concentrations of both drugs. In contrast, RSMs for ifosfamide-doxorubicin pairing displayed a modest decrease in viability output when drug concentrations increased (Fig. 5ai). To validate this interaction, we performed single-drug and combination dose–response assays on five early-passage PDCs to obtain respective IC_50_ values for combination index (CI) calculations based on the Chou-Talalay method. For the combination of pazopanib and AZD5153, the interaction between both drugs showed an increasingly synergistic trend (CI < 1) with increasing drug concentrations across the panel of PDC lines tested. While the CI of pazopanib+ AZD5153 remained in the synergistic range throughout the concentrations tested, in contrast, the CI of ifosfamide+ doxorubicin was modestly reduced at higher concentrations (Fig. 5aii). These results indicated that pazopanib+ AZD5153 is a synergistic and effective combination with superior efficacy compared to ifosfamide+ doxorubicin.

Given the limited availability and proliferative capacity of non-immortalized PDCs, downstream mechanistic work was subsequently performed in a panel of LPS cell lines. Pazopanib-BETi synergy was further confirmed by the Bliss synergy scoring model which revealed synergistic growth inhibition with pazopanib+ AZD5153 pairing but not with ifosfamide+ doxorubicin in a panel of representative STS cell lines (Fig. 5b). We next examined if the combination of pazopanib+ AZD5153 is involved in the induction of apoptosis or alteration of the cell cycle progression in LPS141, DDLPS line displaying strongest combinatorial synergy. Annexin V/PI co-staining showed that co-treatment significantly increased the population of LPS141 cells positively stained for Annexin V and PI, compared to individual drug treatments (Fig. 5c). This was further evaluated by cell cycle analysis with greater accumulation of cells in the G2/M phase following combination treatment as compared to single-drug and control-treated cells (Fig. 5d). Collectively, these findings support Pazopanib-BETi synergy in both patient-derived and cell lines, with the enhanced efficacy of this combination mediated by the induction of cell cycle arrest and apoptosis.

### Pro-apoptotic effects of Pazopanib and AZD5153 are mediated by targeting BRD4-MYC axis

**Fig. 6:**
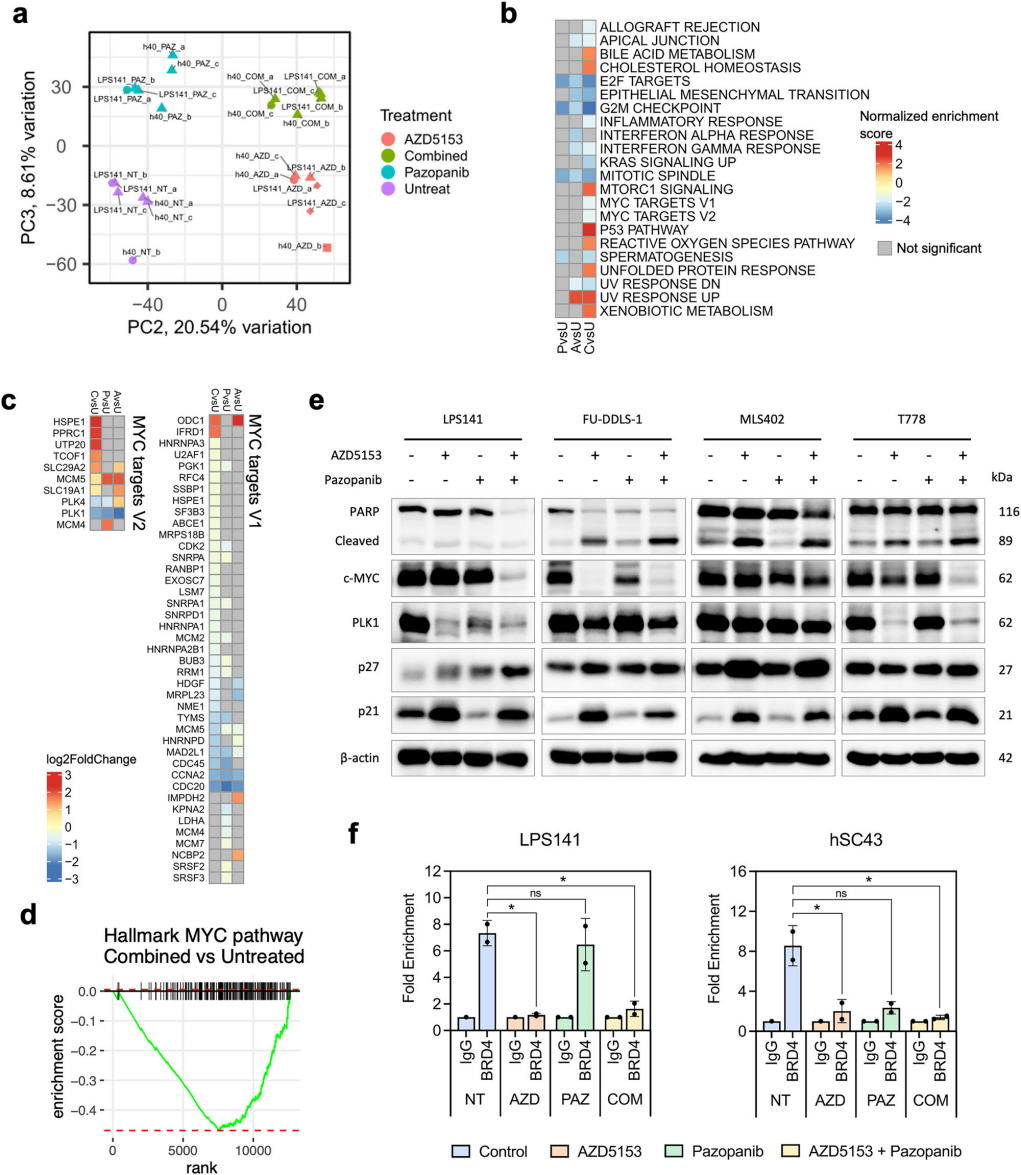
Pazopanib and AZD5153 exhibits pro-apoptotic effects by supressing oncogenic MYC pathways. **a** Principal component analysis (PCA) of log_2_ fold change in gene expression of untreated and treated groups of LPS141 and hSC40 across three biological replicates. **b** GSEA analysis to identify significantly enriched hallmark pathways. Pathways with a fold change (FC) of ≥1 and *P* < 0.05 were considered significantly altered. AvsU, AZD5153 vs Untreated; PvsU, Pazopanib vs Untreated; CvsU, Combined vs Untreated. **c** Heatmap of log_2_ fold change in gene expression of MYC targets 1 and 2 gene class in treated vs untreated groups. AvsU, AZD5153 vs Untreated; PvsU, Pazopanib vs Untreated; CvsU, Combined vs Untreated. **d** Enrichment plots showing negative enrichment of hallmark MYC pathway genes in GSEA Hallmark analysis, as depicted by the profile of the running ES Score and positions of gene set members on the rank-ordered list. AvsU, AZD5153 vs Untreated; PvsU, Pazopanib vs Untreated; CvsU, Combined vs Untreated. **e** Representative immunoblots of PARP apoptotic marker, transcriptional regulator c-MYC, cell cycle kinase PLK1 and its downstream effectors p27 (Kip1) and p21 (Waf1/Cip1), upon treatment with AZD5153 or pazopanib, singly or in combination, for 48 h. **f** Real‐time polymerase chain reaction (RT‐PCR) analysis of MYC promoter regions following chromatin immunoprecipitation (ChIP) assay on LPS141 or hSC43 cells using BRD4 or rabbit IgG antibody. LPS141 cells were treated with 25 μM AZD5153 and/ or 20 μM pazopanib for 24 h while hSC43 cells were treated 80 μM AZD5153 and/ or 40 μM pazopanib for 24 h. Data presented as mean ± SD across two biological replicates. ns not significant; *, *P* < 0.05.

To elucidate the underlying mechanisms of the potentiating effect observed in combination treatment, RNA-seq was performed on DDLPS cell line (LPS141) and established DDLPS PDC (hSC40), untreated, or treated with AZD5153 or pazopanib alone or in combination. After accounting for inherent difference between cell lines (Supplementary Fig. 4), PCA analysis revealed distinct clustering patterns grouped by treatment type, highlighting similar treatment response pathways shared across both cell lines. The combination treatment group clustered more closely with AZD5153, compared to pazopanib group, suggesting that combinatorial effects are largely mediated by treatment effects of AZD5153 (Fig. 6a). We next performed gene set enrichment analysis (GSEA) to identify differentially regulated pathways across treatment types when compared to control group. Apart from canonical pathways that are cell cycle related (E2F targets, G2M checkpoint and P53 pathway), KRAS and MTORC1 signaling (Supplementary Fig. 5) that were differentially enriched, we observed that MYC target gene sets^38^ were negatively enriched with combinatorial treatment as compared to monotherapy with either drug (Fig. 6b). Hallmark GSEA across all DEGs further identified many down-regulated MYC target genes from combination treatment group compared to either monotherapy (Fig. 6c, Supplementary Fig. 6), further supported by significant downregulation as revealed by enrichment plot (*P* < 0.05) (Fig. 6d).

In order to validate sequencing results, we performed immunoblot analysis and showed that co-treatment resulted in induction of apoptosis via PARP cleavage, downregulation of MYC oncoprotein and its downstream target PLK1 cell cycle kinase in a panel of representative cell lines. This was accompanied by upregulation of G1-checkpoint CDK inhibitors p27 and p21, both of which inhibit the activity of cyclin-CDK complexes leading to cell cycle arrest (Fig. 6e). Notably, MYC was most significantly and consistently downregulated with co-treatment across all four cell lines. BRD4 is widely known to occupy and regulate the expression of MYC as a reader protein. As BET inhibitor AZD5153 has been shown to target BRD4 via a bivalent mode of action and decrease c-MYC expression^33^, we conducted BRD4 chromatin immunoprecipitation-qPCR (ChIP-qPCR) assay to investigate if BRD4-mediated control over MYC is altered upon treatment. We performed this analysis in LPS141 and hSC43, an early-passage, fast-growing PDC demonstrated to overexpress MYC. First, qPCR analysis of baseline expression profile further confirmed overexpression of *MYC* in both cell lines. Second, BRD4-mediated *MYC* overexpression was significantly downregulated upon AZD5153 or combinatorial treatment (Fig. 6f). Collectively, these findings indicate that downregulation of oncogenic MYC and MYC-related gene programs contributes to combinatorial synergy, and may be facilitated by the abrogation of BRD4-mediated control over *MYC*.

### Pazopanib and AZD5153 demonstrate anti-tumor activity against DDLPS in vivo

**Fig. 7:**
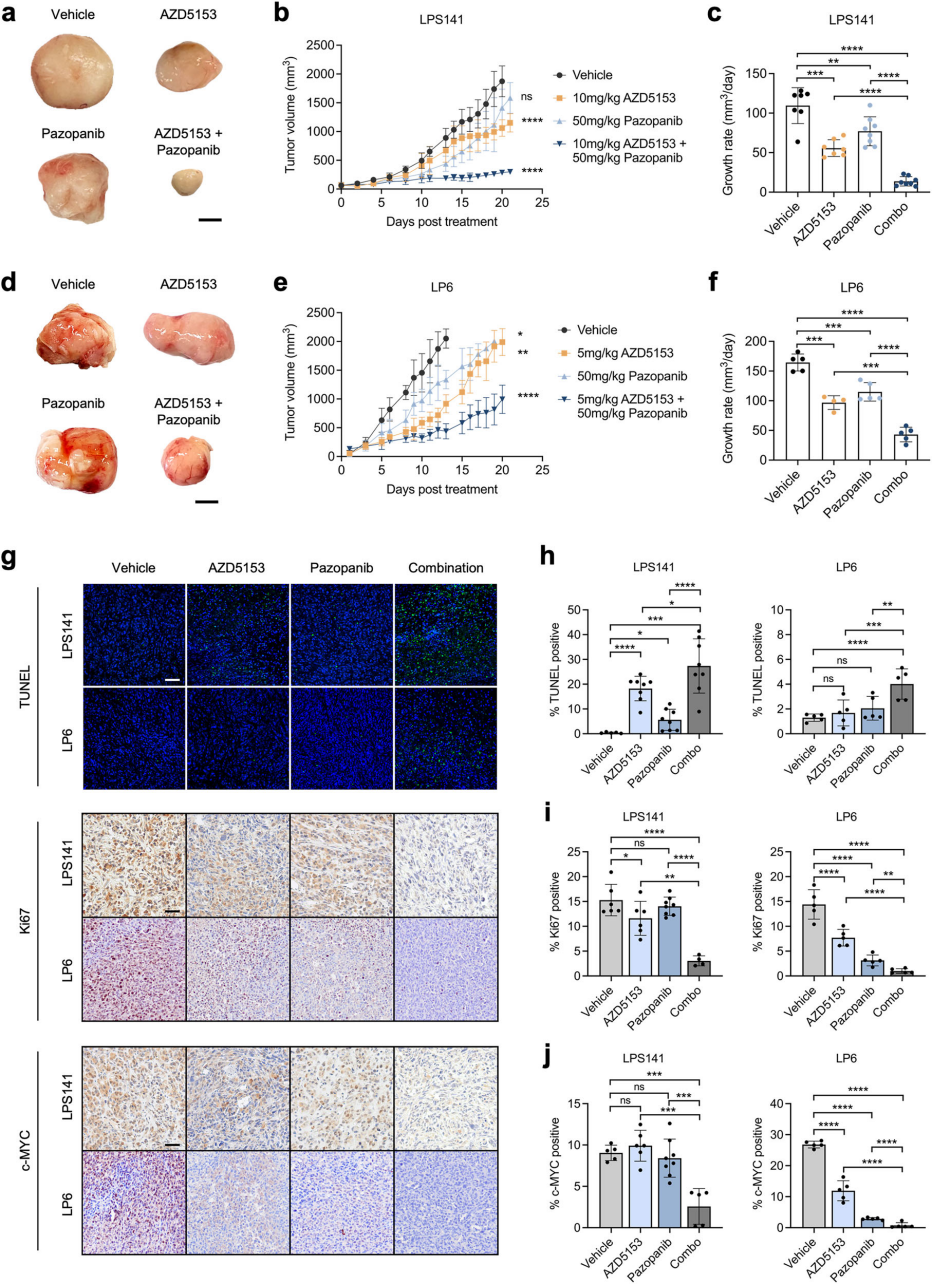
Efficacy of AZD5153 and pazopanib combination against DDLPS mice xenograft. 2.5 × 10^6^ LPS141 or LP6 cells were injected subcutaneously on the right flanks of 5–6 week old, female, NOD-SCID-Gamma (NSG) mice. Mice were sacrificed at endpoint, and tumors were harvested. **a** Representative tumor images, **b** tumor volumes, and **c** growth rates of LPS141 tumors after treatment with vehicle control (0.5% hydroxypropylmethylcellulose and 0.2% Tween 80), AZD5153 (10 mg/kg), pazopanib (50 mg/kg), AZD5153 + pazopanib. Scale bar denotes 0.5 cm. Data presented as mean ± SD, *n* = 5 to 8. ****, *P* < 0.0001; as compared to vehicle at Day 15. **d** Representative tumor images, **e** tumor volumes, and **f** growth rates of LP6 tumors after treatment with vehicle control (0.5% hydroxypropylmethylcellulose and 0.2% Tween 80), AZD5153 (5 mg/kg), pazopanib (50 mg/kg), AZD5153 + pazopanib. Scale bar denotes 0.5 cm. Data presented as mean ± SD, *n* = 5. *, *P* < 0.05; **, *P* < 0.01; ****, *P* < 0.0001; as compared to vehicle at Day 15. **g** Representative immunohistochemical images with accompanying quantification of **h** TUNEL, **i** Ki67, and **j** c-MYC staining following drug treatment. Scale bar = 50 μM. All statistical analysis were performed using two-tailed Student’s *t* test.

To assess the in vivo tumor-suppressive activity of AZD5153 and pazopanib, mice bearing LPS141 or LP6 tumors were treated with AZD5153 or pazopanib as monotherapy or in combination. Combinatorial treatment induced significant reductions in LPS141 tumor volumes (Fig. 7a, b) and tumor growth rates (Fig. 7c) as compared to vehicle and single-drug treatments. Similarly, LP6 tumor bearing mice developed significantly smaller tumors (Fig. 7d, e) and had the lowest growth rates (Fig. 7f) when co-treated with AZD5153 and pazopanib as compared to either agent alone, despite having a higher in vivo growth rate compared to LPS141 tumors. We also measured changes in mice body weight to assess potential treatment-related toxicities and observed no significant loss of body weight following single-drug or combination treatment in both LPS141 and LP6 tumor-bearing mice. This indicates that the dosages used were tolerable.

Apart from impairing LPS tumor growth progression, AZD5153 and pazopanib combination treatment resulted in significantly higher levels of apoptosis in both LPS141 and LP6 tumors as compared to vehicle or single-drug treatments, as shown by TUNEL analysis (Fig. 7g, h). Immunohistochemical analysis of co-treated tumors also revealed significantly lower expression levels of Ki67 proliferative marker than tumors from other treatment groups (Fig. 7g, i). This was accompanied by concomitant decrease in MYC expression in co-treated tumors (Fig. 7g, j), consistent with our findings from in vitro assays. Overall, increased apoptotic response and decreased MYC expression highlight AZD5153 and pazopanib as an effective drug combination in mitigating DDLPS progression in vivo, with significant tumor suppressive activity compared to either drug alone.

## Discussion

A hallmark of STS is genetic, biologic and clinical heterogeneity, which presents particular challenges for accurate diagnosis, prognosis, and treatment. Cytotoxic chemotherapy has remained first-line conventional systemic treatment for decades and the development of more effective targeted options for STS patients has been scant. Given the limited therapeutic options, personalized approaches that allow comprehensive assessment of drug sensitivity or resistance in individual tumors may therefore offer a potential solution to improve treatment outcomes on an individual basis. Accumulation of this information across larger cohorts of these rarer tumors may also allow the identification of therapies that could be effective across subtypes.

Herein, we successfully performed ex vivo QPOP screen in a total of 45 unique primary STS patient samples, and showed improved or concordant patient outcomes that are attributable to QPOP predictions. We next explored QPOP’s ranking function using a panel of standard agents, FDA-approved and experimental drugs for rational selection of active combination regimens across STS histologies and molecular subtypes within a patient-based research system. We revealed an epigenetic-based targeted combination that outperforms clinically-approved regimens, and validated its combinatorial efficacy in a series of in vitro and in vivo assays.

Precision medicine in cancers traditionally involves linking the genomic and functional signatures of patient tumors to prioritize rational treatments that target the driver alterations in each individual’s cancer. This approach often requires the clinical application of omics technologies for which progress has been limited. Most STS patients do not possess any actionable mutations^5^; moreover, this strategy necessitates prior knowledge of established genomic biomarkers of drug responses^39^, which might be lacking in individual cases. To overcome these hurdles, we employed a mechanism-agnostic, quantitative phenotype-based analysis via QPOP in individual tumor samples to rapidly profile patient-specific therapeutic sensitivity and resistance. QPOP bypasses the need for predictive biomarkers to assess drug responses from a functional standpoint. QPOP recommendations were successfully generated for 88% of all samples received and delivered to clinicians within a rapid turnaround time of a week from sample collection to report generation. We further demonstrated concordant QPOP response to clinical outcomes and showed that QPOP can effectively guide clinicians by expanding on, and ranking all available treatment options in patients with limited options. As highlighted in our first patient (Fig. 2), inter-tumor heterogeneity and differential drug response between lesions may exist. However, it is also possible to identify drugs that share a common sensitivity across lesions, as demonstrated with eribulin which showed remarkable clinical efficacy. Nevertheless, the ability to identify effective drugs in rare sarcomas with no other systemic options remains highly valuable in the clinic. Taken together, our study not only represents one of the largest STS cohorts for which ex vivo drug testing was achieved^40^, but also showcased the value of QPOP in the rare disease space for which clinical decisions are hindered by the design of trials to identify effective regimens.

Single agents, usually, are not capable of causing a sustained response and patient benefit is transient. Combination therapy was key to curing over 90% of previously incurable diseases^21^. In applying QPOP for combination discovery, we identified a rational pairing of investigational agent, BETi, and FDA-approved anti-angiogenic, pazopanib, which exhibits stronger combinatorial efficacy compared to standard of care ifosfamide and doxorubicin. We further validated this combination in a panel of PDCs and representative cell lines and demonstrated increased in vitro and in vivo apoptotic response that may be facilitated by targeting the BRD4-MYC axis. A previous study has detailed the involvement of BET proteins in liposarcomagenesis and demonstrated significant elevation of BRD4 in LPS relative to normal fat tissues^41^. The authors further showed high susceptibility towards a panel of BET-targeting agents in LPS, by disrupting oncogenic transcription of *FOSL2*, *MYC*, and *SNAI2*. Our findings therefore corroborate the potent modulation of a clinically relevant on-target biomarker, MYC, by targeting BET protein dependency. Several preclinical studies have demonstrated the efficacy of BRD4 inhibitors in inhibiting tumor growth and metastasis in STS models of various subtypes^34^. Correspondingly, an interesting study by Kurimchak et al. showed that BET inhibitors have limited success as single agents in ovarian cancer due to adaptive kinome reprogramming and therefore require combination therapies simultaneously targeting kinases and BET bromodomain proteins^42^. The authors further revealed that activation of compensatory pro-survival kinase networks overcomes BET protein inhibition, therefore, drug combinations blocking these kinases may prevent or delay the development of drug resistance and enhance the efficacy of BETi therapy. Additionally, other recent studies convincingly demonstrate that BET inhibitors can prevent transcriptional reprogramming triggered by numerous kinase inhibitors, which leads to improved efficacy in preclinical models of solid tumors, such as breast cancer and melanoma^43,44^, thus suggesting the use of BET inhibitors in combination with kinase inhibitors. These discoveries therefore lend support to our observations and provide a rational basis for the investigation of BETi and pazopanib combination in STS. Our study also highlighted the ability of QPOP to pick up on non-genetic mechanisms predominantly conveyed by altered epigenetic states, in addition to mutation-driven mechanisms, that may serve as potential druggable targets. Nonetheless, BETi and their toxicity profiles have been documented in several phase I or II studies for patients with solid tumors and hematologic malignancies^45^. Therefore our approach is to use a specific BRD4i, AZD5153, rather than a BETi which is pan-BRD and more toxic. While early clinical trial data has shown AZD5153 to be generally well tolerated (NCT03205176)^46^, sustained BRD4 inhibition in vivo led to long-term toxicities though phenotypic consequences such as hematologic toxicities were transient and reversible^47^. Clinical management such as intermittent dosing schedules or targeted delivery methods should therefore be explored to minimize long-term systemic toxicities.

Pazopanib, a multitargeted tyrosine kinase inhibitor, is FDA-approved for metastatic STS in the second-line setting^17,48^, with the exception of adipocytic STS or GIST for which it did not meet pre-specified endpoints. Herein, QPOP-derived viability output (one-drug NCV) for pazopanib was consistently low across majority of primary STS patient samples (Fig. 1g), in contrast to palbociclib which displayed higher viability output but demonstrated preclinical activity in DDLPS/WDLPS^49,50^. Although pazopanib was not specifically approved for LPS in the landmark Phase III PALETTE trial, it remains widely used as off-label treatment in LPS given its clinical utility. Our findings, supported by real-world evidence papers^51–53^ and trial data (NCT01506596, NCT01692496), show that pazopanib may in fact, provide therapeutic benefits to select LPS patients. Further investigation into clinical sensitivity of pazopanib in larger, dedicated studies focusing on WD-DDLPS is therefore warranted. Regardless, a major challenge for most patients on single-agent pazopanib is the development of resistance, exacerbated by the lack of predictive biomarkers to guide further therapy^54^. The mechanisms of resistance to multi-kinase drugs such as pazopanib are complex and diverse, and may be intrinsic or acquired^55^. Findings in human synovial sarcoma models has shown that despite strong inhibition of its main PDGFRα/β target, the over activation of compensatory pathways suggests the need for combinatorial partner to restore effective inhibition^56^. Attempts to combine pazopanib with chemotherapy to overcome resistance has been challenging, as the combination was associated with toxicity and did not improve upon the response of either agent^37^. Preclinical studies have demonstrated safety in combining pazopanib with HDAC, mTOR, Her2, or MEK inhibitors, and further suggested pairing of pazopanib with epigenetic inhibitors^57^. Although the efficacy of Pazopanib in combination with a BET inhibitor has never been investigated in the clinical setting, previous studies have suggested that synergistically targeting tyrosine kinase signaling and BRD4 may be useful in the treatment of STS^58^. Targeting the IGF1R/PI3K/AKT pathway has been shown to sensitize Ewing Sarcoma to BET bromodomain inhibitors, inducing a strong synergistic response and potent apoptosis in combination both in vitro and in vivo^59^. Furthermore, JQ1 has been reported to suppress tumor angiogenesis in sarcoma xenograft models of childhood cancer^60^. These results further provide proof-of-concept evidence for combining BET inhibitors with pazopanib in STS.

Considering that doxorubicin has maintained its superiority in OS in clinical trials for over a decade^9–12^, it is interesting that QPOP and subsequent validation work have shown that the combination of BET inhibitor and pazopanib outperforms the standard doxorubicin/ifosfamide regimen. We propose that several unique aspects of the QPOP model and drug combination may explain the enhanced efficacy of this combination. Firstly, QPOP overcomes limitations of conventional drug development and screening strategies that rely on mechanistic assumptions. It provides an unbiased approach that ranks all possible drug-dose permutations from best to worst based solely on experimental data. In this way, QPOP can accurately model complex drug effects and interactions in any given biological system, while eliminating the influence of complex molecular networks or feedback mechanisms that may not be fully understood. This approach is particularly useful for biologically heterogeneous cancers, as demonstrated by our study cohort of 45 patient samples representing at least eight different subtypes, each with distinct treatment histories and clinical responses. Besides, our cohort includes patients with refractory or recurrent disease who progressed on initial lines of treatment, including doxorubicin. Therefore, it is not surprising that QPOP does not prioritize standard regimens as top-ranked, instead identifying other combinations to which patients may potentially respond. Secondly, we propose that the enhanced efficacy of the combination may be attributed to epigenetic plasticity that is dynamic and reversible. There is growing evidence that many STS subtypes are characterized by widespread epigenetic dysregulation^34,61^. By modulating gene expression, BET inhibitors can shift or restore the balance in tumor cells toward sensitivity to pazopanib, potentially reversing some of the adaptations that lead to resistance. In elucidating the cellular mechanisms of synergy, we observed consistent and synergistic downregulation of MYC expression at both the mRNA and protein levels with combinatorial treatment. While BRD4 is widely recognized for its role in occupying and regulating *MYC* expression as a reader protein, the mechanisms by which pazopanib modulates MYC have been documented in only a few studies^62–64^. Although the exact mechanisms remain unclear, we propose that the inhibition of growth factor signaling and the targeting of receptors such as VEGFR, PDGFR, and c-KIT may reduce signals that promote MYC expression and activity. Additionally, pazopanib could directly or indirectly reduce *MYC* transcriptional activity through downstream effects on other signaling pathways, such as MAPK or PI3K, which regulate *MYC* expression.

Overall, our study demonstrates feasibility for the QPOP platform to identify drug combinations against STS that is otherwise resistant to standard of care, and supports the utility of phenotypic-driven drug discovery for personalized medicine in this setting. Nonetheless, our study contained several limitations. Firstly, due to our small sample size, there was a limited pool of patients available for comparing clinical outcome concordance with QPOP-defined outcomes. Future work will include an expanded clinical trial to investigate clinical benefit of QPOP-guided therapies, as well as the combination of BETi and pazopanib, in larger STS cohorts. Incorporation of single-cell analysis to evaluate bulk or sub-clonal phenotypic effects may serve to identify and integrate key modulators in signaling pathways. In depth sequencing and molecular profiling of exceptional responders in clinical trials would also be instructive for future patients especially in rare diseases like STS. By combining sequencing and deep molecular profiling with QPOP data, an improved approach to functional precision testing may further support patient-specific treatment guidance and identify attractive subtype-specific targets^65^. Within an expanded trial, a cumulative and collaborative workflow of pairing functional and molecular datasets will serve as the foundation for refining individualized treatments and the development of predictive biomarkers. Secondly, because STS are rare tumors, it is difficult to prospectively accumulate clinical data from patients with different histologic or molecular subtypes and evaluate subtype-specific drug efficacy. Our patient samples were consecutively obtained solely based on sufficient cell count and not selected for any specific attribute. Whether the combination of BETi and pazopanib is broadly effective or subtype-specific remains to be explored. To this end, we postulate that our patients are representative of those referred to tertiary care centers and therefore our results are relevant to the general STS population. Nonetheless, we aim to further interrogate BET protein dependency and its mechanistic connections to aberrant LPS transcriptional programs in cell line and patient-derived models. Similarly, this work can be paired with multi-omics analysis for eventual biomarker and proteomics marker discovery. These findings can then be validated back in ex vivo PDCs, with predictive biomarkers therefore serving as a diagnostic readout to facilitate patient stratification and accelerate clinical decision making.

In summary, we evaluated combinatorial drug response in the largest reported ex vivo drug testing cohort of STS patient samples. High concordance between ex vivo drug testing results and clinicoradiological evidence demonstrates the feasibility of a patient-based drug analytic approach to identify therapeutic options in rare cancers that otherwise has no clear treatment guidelines or effective salvage options. QPOP’s combination therapy ranking function prioritized an epigenetic-based targeted combination, which displayed enhanced synergy both in vitro and in vivo, and superior to standard of care ifosfamide and doxorubicin. We further showed that BETi-pazopanib synergy may be facilitated by the abrogation of BRD4-mediated control over *MYC*. Overall, our work supports the utility of phenotypic-driven drug discovery for personalized medicine and provides preclinical evidence for the further development of BETi-pazopanib as an alternative treatment option for STS.

## Methods

### Patient tissue and clinical data collection

Written informed consent was obtained from all patients for use of biospecimens, and consent for publication was obtained from the patients included in the case reports. The study adhered to all relevant ethical guidelines, including the Declaration of Helsinki. Tissue collection and consent protocols were governed by ethics approval from the SingHealth Centralized Institution Review Board (CIRB-2018/3182). QPOP analysis of patient samples was performed in accordance with ethics approval from NUS Institutional Review Board (H-18-032).

### Dissociation and establishment of patient-derived cells (PDC)

Freshly excised tumor samples were digested with a GentleMACS dissociator in warm RPMI-1640 medium (Biowest) with 100 μg/mL LiberaseTM (Sigma Aldrich, Singapore), followed by incubation on a shaker at 70 rpm in a cell culture incubator for 2 h. The cell mixture was passed through a 70 μm mesh, and isolated PDCs were used immediately for drug treatment, or cultured in RPMI-1640 medium supplemented with 20% fetal bovine serum (FBS; Gibco, USA), 100 U/mL penicillin/streptomycin (Gibco, USA). PDCs were considered established when they achieve logarithmic growing phase beyond the third passage.

### Ex vivo QPOP drug treatment and analysis

A 12 drug-three dosage (IC_0_, IC_10_, IC_20_) QPOP was performed in this study. The drug panel included cytotoxic chemotherapy agents (ifosfamide, doxorubicin, gemcitabine, paclitaxel, eribulin, and trabectedin), epigenetic drugs (HDAC, DNMT, and BET inhibitors) as well as the CDK inhibitor, (palbociclib) and multi-tyrosine kinase inhibitors (pazopanib and ponatinib). Single-drug dose response assay was performed in parallel to QPOP drug treatment. Dissociated PDPCs were screened against log dose concentrations of the panel of 12 drugs for 48 h to establish their respective dose–response curves and half-maximal inhibitory concentrations (IC_50_). For QPOP combinatorial drug treatment, an orthogonal array composite design (OACD) was used to determine the 155 experimental combinations necessary for sufficient factor screening and model fitting, and tested in technical duplicates^66^. Drugs were dispensed with the aid of a digital dispenser (D300e, Tecan) and cell viability was measured 48 h post drug treatment using CellTiter-Glo (CTG) Luminescent Cell Viability Assay (Promega) according to manufacturer’s instructions. The normalized cell viability scores were used as phenotypic inputs for QPOP second-order regression analysis as described by Rashid et al.^26^. QPOP then generates corresponding viability output values for all possible drug permutations, for which all two-drug combinations were ranked on. Quality assessment of the assay was evaluated using the Z-factor (Z’)^67^ and strictly standardized mean difference scores^68^. Parabolic response surface maps indicating interaction between any two drugs were generated based on QPOP regression analysis using MATLAB (MathWorks).

### Cell culture

T778 is a well-differentiated liposarcoma cell line (WDLPS) obtained from American Type Culture Collection (ATCC, Manassas, VA). FU-DDLS-1, a de-differentiated liposarcoma (DDLPS) cell line, was a gift from Dr. Nishio^69^, while LPS141 and LP6 are DDLPS cell lines provided by Dr Christopher DM Fletcher^70^. MLS402 (myxoid liposarcoma; MLPS) cell line was generated by Dr. Pierre Åman^71^. All the aforementioned cell lines were cultured in RPMI-1640 medium (Gibco) supplemented with 10% FBS and penicillin/streptomycin (100 U/mL). Adipose mesenchymal stem cell ASC52telo (hTERT immortalized adipose-derived mesenchymal stem cell; ASCtelo) was cultured in mesenchymal stem cell basal medium supplemented with mesenchymal stem cell growth Kit (ATCC). All cell cultures were maintained at 37 °C under a humidified atmosphere with 5% CO_2_.

### Determination of half-maximal inhibitory concentration (IC_50_) of drugs

Cells were seeded in 384-well plates (2500 cells/well) 24 h prior to drug treatment. Drugs were prepared in serial dilutions that ranged from 0.0001 to 100 μM and cells were treated in technical quadruplicates for 48 h. Cell viability was then measured using CTG assay (Promega). IC_50_ values of individual drugs were determined using Prism 9 software (GraphPad) by fitting normalized cell viability values into sigmoidal dose–response curves.

### Validation of drug combinations

To validate the top-ranked drug combinations determined by QPOP, we employed the use of mathematical models to quantify the combination effect of two drugs and provide better interpretation and assess reproducibility of QPOP-derived experimental data. In the median-effect (Chou-Talalay) method, single-drug and combination dose-response assays were performed to determine the IC_50_ values of each drug as monotherapy and in combination. The ratio of drug dosages between the two drugs was determined by the dosages used in QPOP and kept constant across the range of concentrations treated. Cell viability was then measured using CTG assay (Promega) 48 h post drug treatment and the combination index (CI) values were calculated according to the Chou–Talalay method^72^. Additionally, the Bliss independence model was used to compute the degree of drug synergy by categorizing combinations as synergistic, antagonistic or additive. Serial dilutions of either drug were prepared in deep well plates and added to cells in a 5 × 5 matrix of varying drug doses, with technical quadruplicates. Cell viability was measured after 48 h using CTG Assay (Promega). Bliss synergy scores were computed by inputting normalized cell viability values into SynergyFinder 2.0 application^73^. Based on deviation of observed and expected responses in the Bliss independence model, the drug combination interaction is classified as synergistic (>10) or antagonistic (<−10).

### Apoptosis assay

Following 48 h of drug treatment, cells were harvested and apoptosis levels were determined using the BD Pharmingen™ FITC Annexin V Apoptosis Detection Kit as per manufacturer’s instructions (BD Biosciences). Briefly, cells were stained with FITC Annexin V antibody and Propidium Iodide (PI) and percentage of cells undergoing apoptosis was measured by flow cytometry (BD LSRII, BD Biosciences). At least 10,000 events were analyzed for each sample. Quantification of percentage of apoptotic cells was performed using FlowJo software.

### Cell cycle analysis

Following 24 h of drug treatment, cells were harvested, washed in PBS and fixed with cold 70% ethanol for at least an hour at 4 °C. Cells were then incubated with RNAse A (100 μg/ml) and propidium iodide (50 μg/ml) for 10 min. DNA content distribution across the different phases of the cell cycle was measured using flow cytometry (BD LSRII) and analyzed with FlowJo software.

### Western blot

Cells were pelleted and washed with cold phosphate buffered saline (PBS) twice before being lysed in RIPA lysis buffer (ThermoFisher Scientific) containing both phosphatase (PhosSTOP; Roche) and protease inhibitors (cOmplete^TM^ Protease Inhibitor Cocktail; Roche). The lysates were collected, centrifuged at 12,000×*g* for 10 min, and respective protein concentrations were determined with a bicinchoninic acid protein (BCA) assay kit (Pierce, Iselin, NJ, USA). Equal amounts of protein lysates were resolved by SDS-PAGE, transferred onto PVDF membranes and serially stained with primary and secondary HRP-conjugated antibodies according to standard procedures. Protein bands were then detected via chemiluminescence using the ChemiDoc Imaging system (Bio-Rad). Primary antibodies used include PARP (Cell Signaling Technology, #9542), c-MYC (Abcam, ab32072), PLK1 (Cell Signaling Technology, #4513), p27 Kip1 (Cell Signaling Technology, #3686), p21 Waf1/Cip1 (Cell Signaling Technology, #2947) and β-actin (Sigma, #A5441).

### Chromatin immunoprecipitation (ChIP) and quantitative PCR

Cells were washed with cold PBS twice, crosslinked with 1% (v/v) formaldehyde (Pierce) for 15 min and quenched with 0.125 M glycine. The fixed cells were lysed and sonicated for 15 cycles (15 s ON/ 30 s OFF) at 30% amplitude using EpiShear Probe Sonicator (Active Motif). In total, 25 μg of sheared chromatin was incubated with 5 μg of antibodies overnight at 4 °C under rotation. In total, 25 μL of pre-equilibrated Dynabeads™ Protein G (Invitrogen, 10003D) was added to the ChIP reactions the next day and left to conjugate for 2 h at 4 °C. Beads were washed thoroughly, eluted in fresh elution buffer (1% SDS, 0.1 M NaHCO_3_) and de-crosslinked overnight at 65 °C. DNA samples were treated with RNase A and Proteinase K, purified with the QIAquick PCR purification kit (Qiagen), followed by qPCR analysis using the QuantiNova SYBR Green PCR Kit (Qiagen) on QuantStudio 5 Real-Time PCR Systems (Applied Biosystems) with primers described in Supplementary Table 1.

### In vivo drug treatment

All animal experiments were approved and performed according to the National University of Singapore Institutional Animal Care and Use Committee (IACUC) guidelines. A 5–6 weeks old female NOD/SCID/IL2rγnull (NSG) mice were purchased from InVivos, Singapore, and acclimatized for one week before the start of the experiment. 2.5 × 10^6^ LPS141 or LP6 cells were injected into the flanks of NSG mice. The mice were then randomized into 4 groups (*n* = 5 to 8 per group): vehicle control (0.5% hydroxypropylmethylcellulose and 0.1% Tween 80), AZD5153 (5 or 10 mg/kg), pazopanib (50 mg/kg) and AZD5153 + pazopanib. One week after tumor inoculation, treatment was administered daily by oral gavage. Tumor growth was assessed by measuring tumor volume, which is defined as *V* = *π*/6 × *A*2 × *B*, where *A* is the smallest superficial diameter and *B* is the largest superficial diameter. All mice were terminated following 21 days of drug treatment, or when tumor volume reaches endpoint of 2000 mm^3^. Tumor growth rate was calculated based on the difference between the first and the last tumor volume measured over the total number of days of treatment.

### Immunohistochemical staining

Harvested tumor tissues were fixed in 4% paraformaldehyde overnight, embedded in paraffin, and sectioned onto glass slides. Tissue sections of 4 μM thickness were stained using standard immunohistochemical procedures with Ki67 (Abcam, 1:500) or c-MYC (Abcam, 1:300) antibodies. Image acquisition was performed using Olympus BX43 and DAB quantification was analyzed using ImageJ software. At least 10 fields of 20× magnification were randomly selected per sample for analysis. Tissues were also subjected to serial staining with haematoxylin and eosin (H&E) for histological analysis.

### TUNEL assay

To determine apoptosis in DDLPS tumors, terminal deoxynucleotidyl transferase- mediated dUTP nick-end labeling (TUNEL) assay was performed using ApopTag® Fluorescein In Situ Apoptosis Detection Kit (Millipore) according to manufacturer’s instructions. Tissue sections of 4 μM thickness were incubated with Terminal Deoxynucleotidyl Transferase (TdT) in a humidified chamber at 37 °C for 1 h, and then incubated with anti-digoxigenin conjugate and peroxidase substrate. Tissues were counterstained with DAPI before image acquisition using the Axio Imager M2. At least 10 fields of 20× magnification were randomly selected per sample for analysis. Quantification of TUNEL-positive cells was analyzed using ImageJ software.

### RNA sequencing and analysis

Total RNA was extracted from LPS141 or hSC40 untreated, treated singly or combination. In total, 5 × 10^6^ LPS141 or hSC40 cells were seeded in 10 cm dishes, allowed to adhere and recover for 24 h prior to drug treatment. Following 48 h of drug treatment, total RNA was extracted and purified with RNeasy Mini kit (Qiagen). RNA concentration was determined by Nanodrop 1000 Spectrophotometer (ThermoFisher Scientific). Extracted RNA was converted into RNA sequencing library using the NEBNext® Ultra™ Directional RNA Library Prep Kit (NEB) using manufacturer’s instructions. Whole transcriptome sequencing of cell lines was performed on the Illumina NovaSeq platform (Novogene, Singapore) using the standard Illumina RNA-seq protocol. Cutadapt (3.4) was used to perform quality and adapter trimming. STAR (2.7.10a) was used as the aligner, and RSEM (1.3.1) was used as the quantification tool to generate the count matrix. R (4.3.1) was used for downstream post processing analyses. For differential expression gene analysis and batch correction, edgeR (4.0.9) was used. Genes with <10 counts were discarded before scaling factors to convert raw library sizes to a normalized effective library sizes was carried out. Subsequent analyses were done using PCAtools (2.14.0), clusterProfiler (4.10.1), fgsea (1.28.0) and msigdbr (7.5.1). Heatmaps were visualized using pheatmap (1.0.12).

### Statistical analysis

All experiments were performed in at least biological triplicates unless otherwise stated, with data presented as mean ± standard deviation (SD). Student’s two-tailed *t* test was used for the comparison of two independent groups. A *P* value of <0.05 was accepted as statistically significant. Prism 9 software (GraphPad) was used for data analysis.

## Supplementary information

Supplementary Information

## Data Availability

The phenotypic drug sensitivity data supporting the findings of this study are available upon reasonable request from the corresponding author. The RNA sequencing data have been deposited to the Gene Expression Omnibus (GEO) repository (NCBI) under the accession number: GSE282752.

## Code availability

The detailed code for the QPOP analyses is unavailable as the software, Optim.AI™, is proprietary to KYAN Technologies.
