# Supplementary material for: Functional combinatorial precision medicine for predicting and optimizing soft tissue sarcoma treatments
Source: NPJ Precis Oncol. 2025 Mar 22;9:83. doi: 10.1038/s41698-025-00851-7 (PMC11929909; doi:10.1038/s41698-025-00851-7)

SUPPLEMENTARY INFORMATION

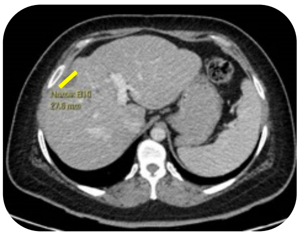

Dec 2022

Supplementary Figure 1: Stable response to eribulin in liver lesion of SFT patient. Computed tomography (CT) imaging of the dominant liver metastasis in December 2022 following of eribulin treatment in May 2022.

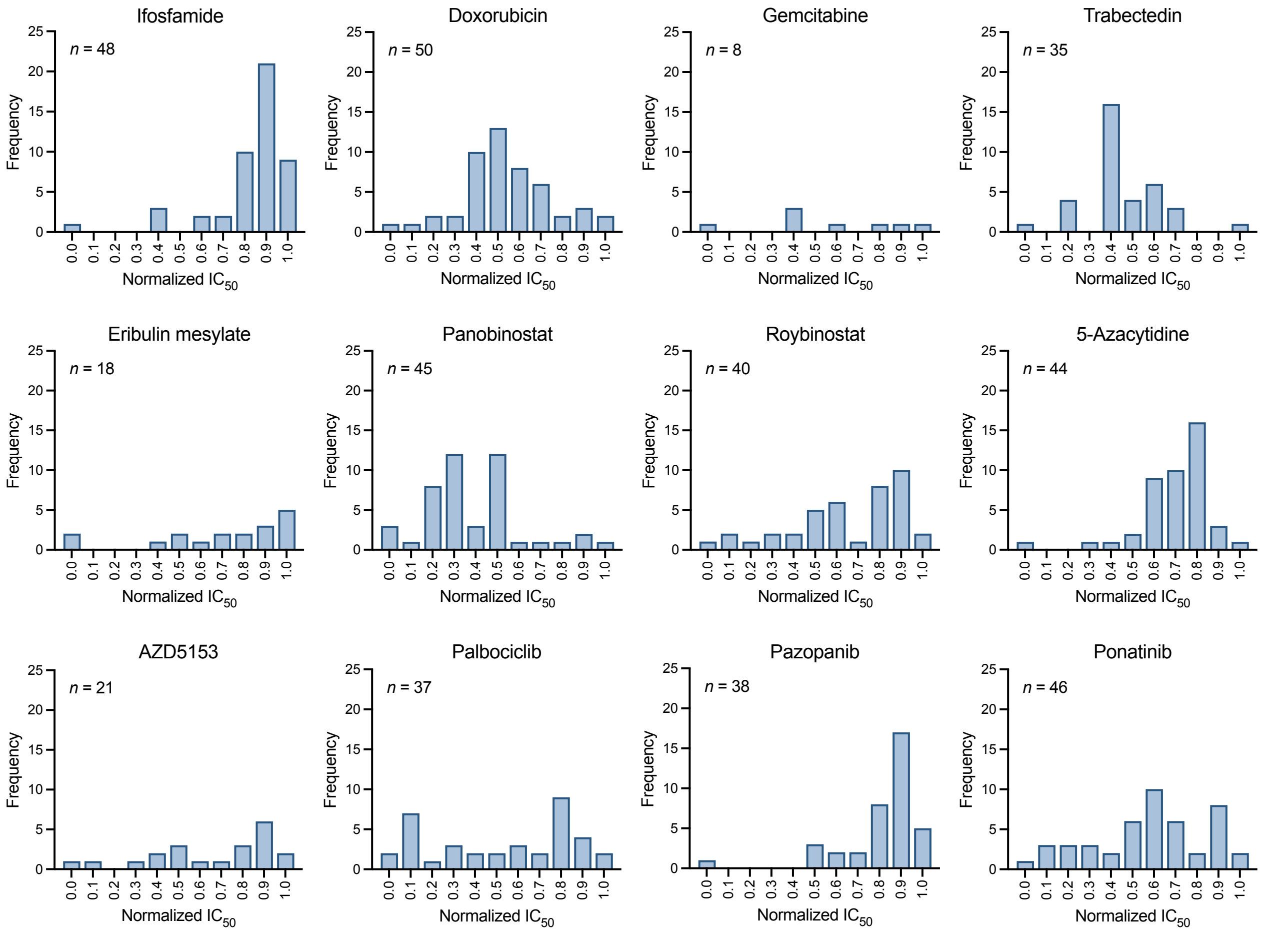

Supplementary Figure 2: Overall distribution patterns of IC_{50} values across 12 drugs. Drug sensitivities for each drug were normalized into a range from 0.0 to 1.0 and the distribution of frequencies for each interval of 0.1 is plotted in a histogram. Overall, there is a wide range of distribution patterns in the patient cohort. Across all drugs, we observed wide interindividual variability in drug sensitivity, with an average coefficient of variation of 41.9% (range= 23.4% to 59.6%).

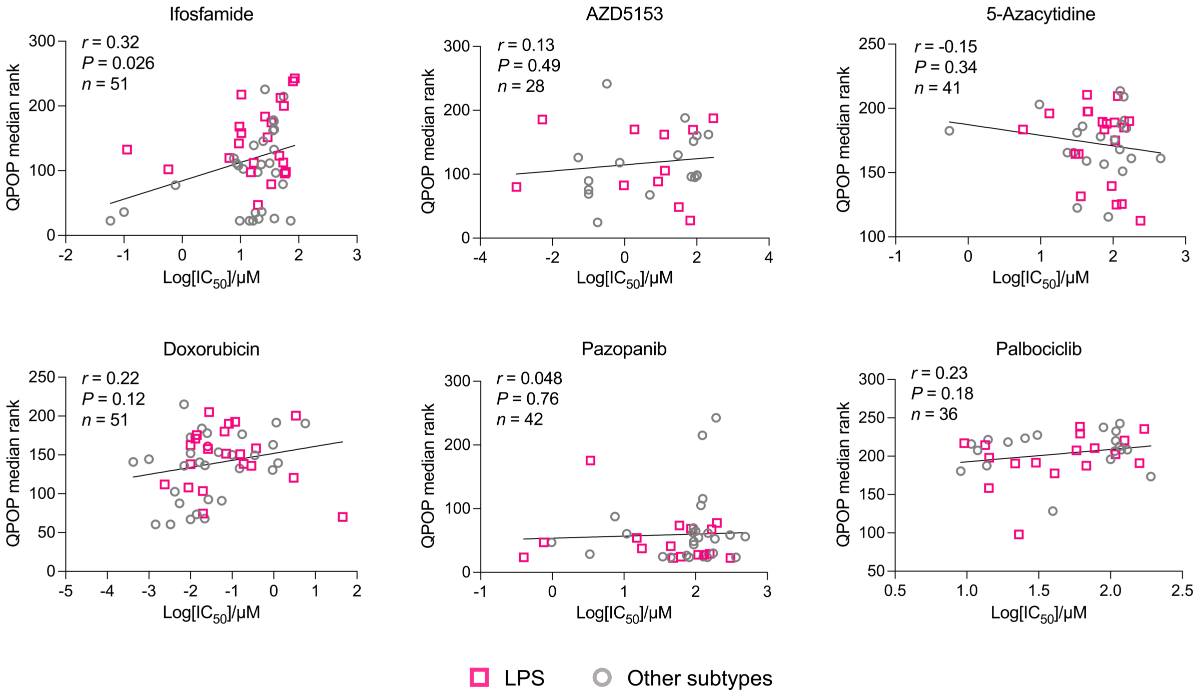

Supplementary Figure 3: Sensitivity to single drug versus combination therapy. Correlation between single drug sensitivities and QPOP drug combination rankings for six drugs in which IC_{50} values could be determined and followed a normal distribution. Scatter plots showing correlation between logarithmic IC_{50} values of single drugs (*x*-axis) and QPOP median rank (*y*-axis). "QPOP median rank" is obtained from the median value of all possible two-drugs combinations containing the drug within the drug panel for each individual patient. A lower IC_{50} or QPOP median rank value represents greater cell killing. Pearson correlation coefficient (*r*), *p*- and *n*-values shown represent correlation analysis for the overall cohort (LPS and non-LPS).

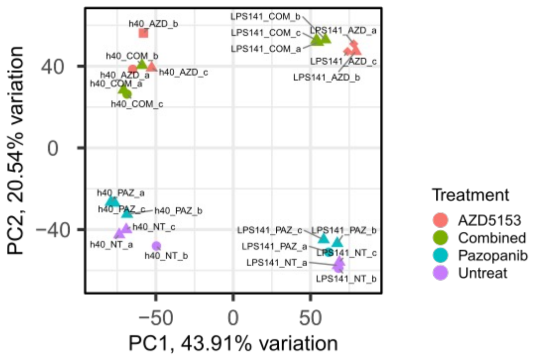

Supplementary Figure 4: Principal component analysis (PCA) showing variance due to inherent difference between cell lines. PCA of log_2 fold change in gene expression of untreated and treated groups of LPS141 and hSC40 across 3 biological replicates.

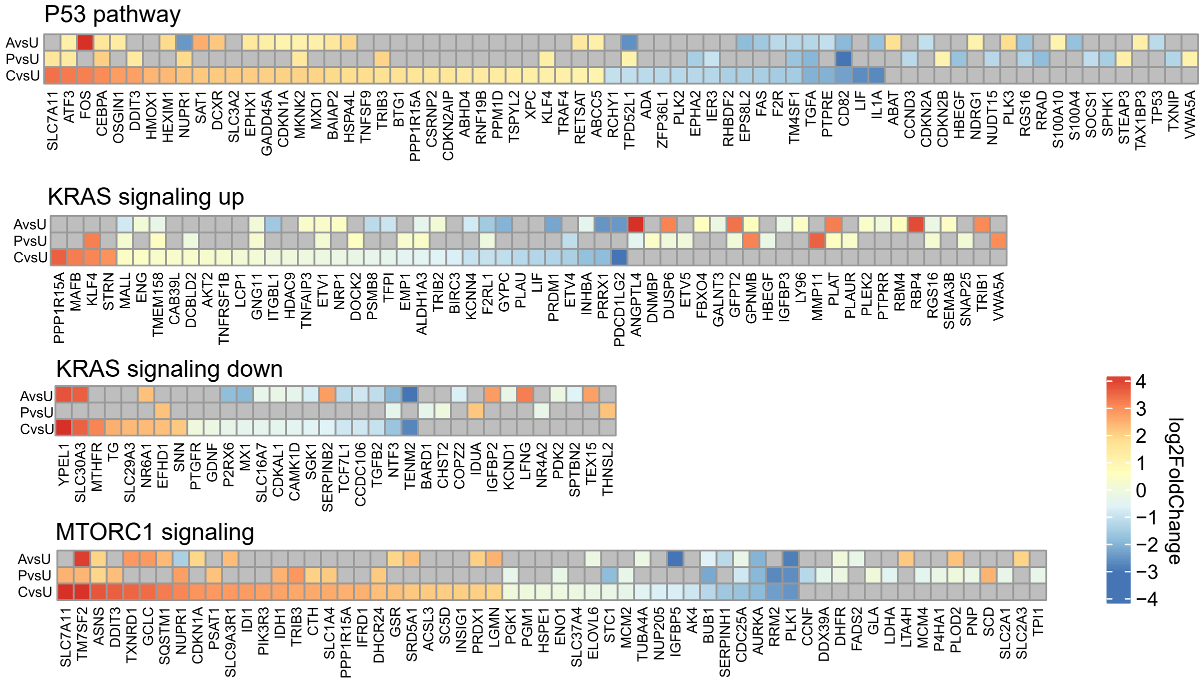

Supplementary Figure 5: Gene set enrichment analysis (GSEA) results of treatment groups vs control comparisons. GSEA Hallmark analysis showing enriched gene sets associated with P53 pathway, KRAS and MTORC1 signaling.

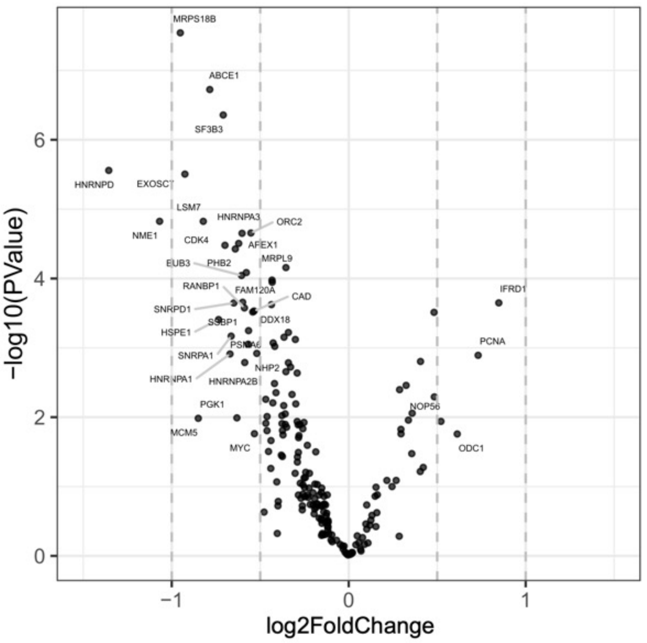

Supplementary Figure 6: Analysis of differentially expressed genes (DEGs) across LPS141 and hSC40 in combinatorial treatment group vs control. Volcano plot showing statistical significance of differential gene expression data (adjusted *p*-value) versus magnitude of expression change (log_2FC).

Supplementary Table 1: Primer sequence for BRD4 ChIP-qPCR analysis.

Gene	Direction (5’ → 3’)	Primer sequence
MYC	Forward	TGCGGGCGTCCTGGGAAG
	Reverse	GTGGATGCGGCAAGGGTTG

Uncropped blots for Figure 6e

PARP (89/ 116 kDa)

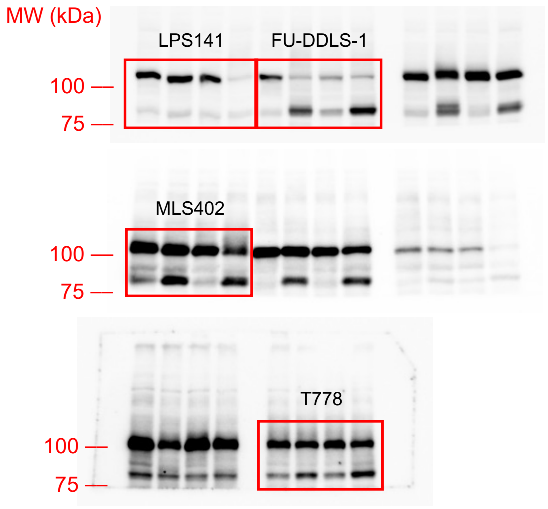

c-MYC (62 kDa)

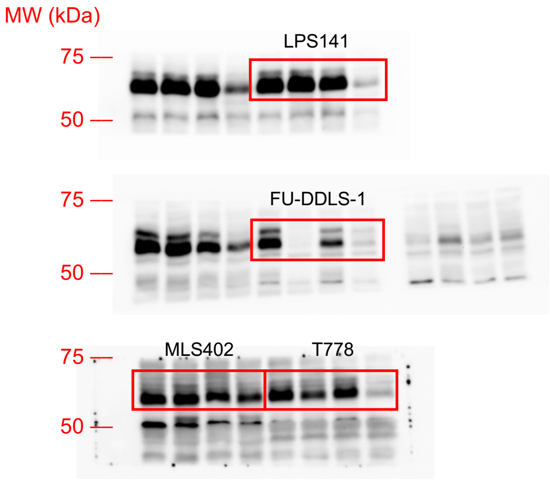

PLK1 (62 kDa)

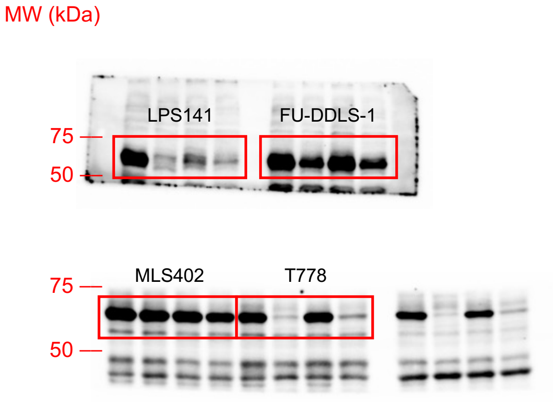

p27 (27 kDa)

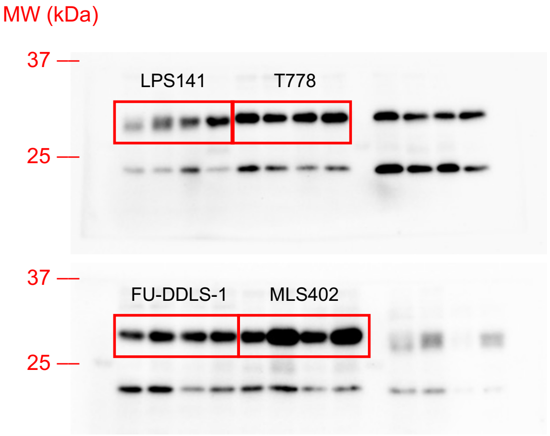

p21 (21 kDa)

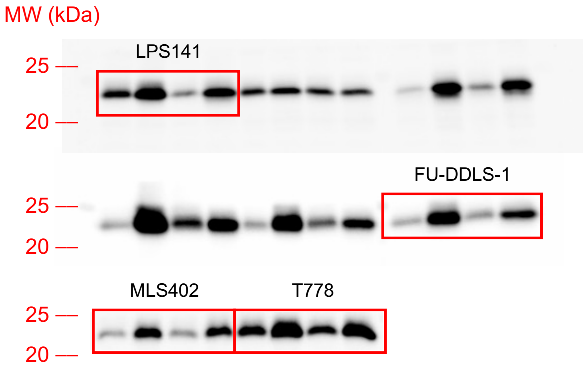

β-actin (42 kDa)

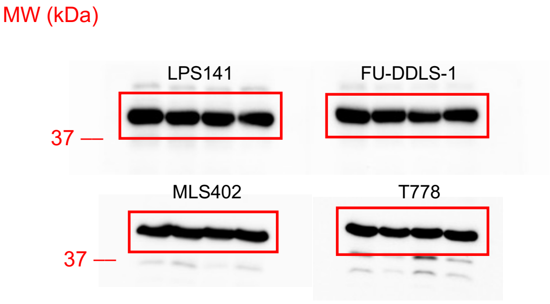

Supplement: Supplementary file 1 — Supplementary Information [file 41698_2025_851_MOESM1_ESM.pdf]
